# Supplementary material for: The Small RNA Universe of Capitella teleta
Source: Front Mol Biosci. 2022 Feb 25;9:802814. doi: 10.3389/fmolb.2022.802814 (PMC8915122; doi:10.3389/fmolb.2022.802814)
Supplement: Supplementary file 1 [file DataSheet1.ZIP › Supplement/known/CAPTEscaffold_158_9961.pdf]

Provisional ID	:	CAPTEscaffold_158_9961
Score total	:	498230.8
Score for star read(s)	:	3.9
Score for read counts	:	498220.6
Score for mfe	:	1.7
Score for randfold	:	1.6
Score for cons. seed	:	3
Total read count	:	977250
Mature read count	:	976010
Loop read count	:	0
Star read count	:	1240

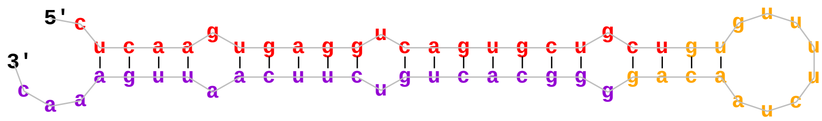

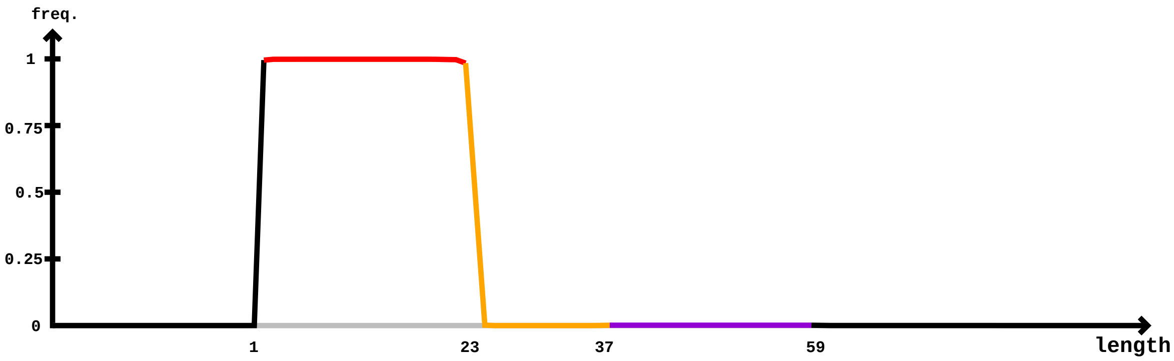

Mature Star

```
5'- uuugucuagcgucucgcugucucaagugaggucagugcugcuguguuuucuaacaggggcacugucuucaauugaaaccguuggaccggaacucaccaucaauuucacuuuc   -3'   obs
    uuugucuagcgucucgcugucucaagugaggucagugcugcuguguuuucuaacaggggcacugucuucaauugaaaccguuggaccggaacucaccaucaauuucacuuuc         exp
    uuugucuagcgucucgcugucucaagugaggucagugcugcuguguuuucuaacaggggcacugucuucaauugaaaccguuggaccggaacucaccaucaauuucacuuuc         known
    ...((((((((.......((.((((.(((((.(((((((.((((........)))).))))))).))))).)))).)))))))))).((......))...............     reads   mm   sample
    .uugucuagcgucucgcugu............................................................................................     2       0    seq
    ..ugucuagcgucucgcugu............................................................................................     3       0    seq
    ..................ucucaagugaggucagugcugcu.......................................................................     3       0    seq
    ..................Ccucaagugaggucagugcugcu.......................................................................     64      1    seq
    ..................Acucaagugaggucagugcugcu.......................................................................     1       1    seq
    ....................cucaagAgaggucagugc..........................................................................     2       1    seq
    ....................cucaagugaggucagugG..........................................................................     2       1    seq
    ....................cucaagugaggucagugc..........................................................................     265     0    seq
    ....................cucaagAgaggucagugcu.........................................................................     2       1    seq
    ....................cucaagugaggucagugcu.........................................................................     760     0    seq
    ....................cGcaagugaggucagugcu.........................................................................     1       1    seq
    ....................cucaagugaCgucagugcu.........................................................................     1       1    seq
    ....................cucaagugaggucagugcG.........................................................................     2       1    seq
    ....................cucaagugaggucagugcA.........................................................................     3       1    seq
    ....................cucaaCugaggucagugcu.........................................................................     1       1    seq
    ....................Uucaagugaggucagugcu.........................................................................     1       1    seq
    ....................cucaagugaggucagAgcu.........................................................................     1       1    seq
    ....................cuAaagugaggucagugcu.........................................................................     2       1    seq
    ....................cucaagugagUucagugcu.........................................................................     1       1    seq
    ....................cucaagugaggucagugcug........................................................................     546     0    seq
    ....................cAcaagugaggucagugcug........................................................................     1       1    seq
    ....................cucaagugaggucagugcuA........................................................................     7       1    seq
    ....................cucaagugaggucagugcuC........................................................................     1       1    seq
    ....................cucaaguAaggucagugcug........................................................................     1       1    seq
    ....................cucaagAgaggucagugcug........................................................................     1       1    seq
    ....................cucaagugaggucagugcuU........................................................................     4       1    seq
    ....................cucaagugagguUagugcug........................................................................     1       1    seq
    ....................cucaagugaggucGgugcugc.......................................................................     2       1    seq
    ....................cucaagugaggucaguUcugc.......................................................................     1       1    seq
    ....................cucaagugaggucagugUugc.......................................................................     1       1    seq
    ....................cucaagAgaggucagugcugc.......................................................................     16      1    seq
    ....................Aucaagugaggucagugcugc.......................................................................     5       1    seq
```

Mature **Star**

```
uuugucuagcgucucgcugucucaagugaggucagugcugcuguguuuucuaacaggggcacugucuucaauugaaaccguuggaccggaacucaccaucaauuucacuuuc
....................cucaaguAaggucagugcugc.......................................................................  11      1  seq
....................cucaagugaggucagAgcugc.......................................................................  13      1  seq
....................cucaagugaggucaguAcugc.......................................................................  2       1  seq
....................cucaagCgaggucagugcugc.......................................................................  1       1  seq
....................cucaagugaggucagugcuUc.......................................................................  1       1  seq
....................cucaagugaggucagugcugc.......................................................................  12331   0  seq
....................cucaagugaggucagugcCgc.......................................................................  1       1  seq
....................cucaagugaggucagugcAgc.......................................................................  3       1  seq
....................cucaagugUggucagugcugc.......................................................................  3       1  seq
....................cCcaagugaggucagugcugc.......................................................................  4       1  seq
....................cucaagGgaggucagugcugc.......................................................................  1       1  seq
....................cucaaguUaggucagugcugc.......................................................................  2       1  seq
....................cucaagugaggucagugAugc.......................................................................  3       1  seq
....................cucaagugaggucagugcGgc.......................................................................  2       1  seq
....................cucGagugaggucagugcugc.......................................................................  2       1  seq
....................cNcaagugaggucagugcugc.......................................................................  1       1  seq
....................cAcaagugaggucagugcugc.......................................................................  13      1  seq
....................cucaagugagCucagugcugc.......................................................................  1       1  seq
....................cucaagugaggucagugcugA.......................................................................  8       1  seq
....................cucaagugaggucUgugcugc.......................................................................  1       1  seq
....................cucaagugGggucagugcugc.......................................................................  1       1  seq
....................cucaagugagguAagugcugc.......................................................................  2       1  seq
....................cucaagugaggucagGgcugc.......................................................................  1       1  seq
....................cuGaagugaggucagugcugc.......................................................................  1       1  seq
....................cGcaagugaggucagugcugc.......................................................................  4       1  seq
....................cucaagugaggucagugcugU.......................................................................  47      1  seq
....................cucaagugaAgucagugcugc.......................................................................  8       1  seq
....................cucaagugaggGcagugcugc.......................................................................  1       1  seq
....................cucaagugagguUagugcugc.......................................................................  2       1  seq
....................cucaaUugaggucagugcugc.......................................................................  1       1  seq
....................cucaagugaggucagugcugG.......................................................................  2       1  seq
....................cucaagugaCgucagugcugc.......................................................................  1       1  seq
....................cucaagugaggucaUugcugc.......................................................................  1       1  seq
....................cuAaagugaggucagugcugc.......................................................................  5       1  seq
....................cucaUgugaggucagugcugc.......................................................................  1       1  seq
....................cucaagugaggCcagugcugc.......................................................................  3       1  seq
....................cucaGgugaggucagugcugc.......................................................................  2       1  seq
....................Uucaagugaggucagugcugc.......................................................................  13      1  seq
....................cuUaagugaggucagugcugc.......................................................................  10      1  seq
....................cucUagugaggucagugcugc.......................................................................  1       1  seq
....................cucaagugaggucagCgcugc.......................................................................  1       1  seq
....................cucaagugaggAcagugcugc.......................................................................  5       1  seq
....................cucaagugagAucagugcugc.......................................................................  1       1  seq
....................cucaagugaggucagugcuCc.......................................................................  1       1  seq
....................cucaaNugaggucagugcugcu......................................................................  11      1  seq
....................cucaagugaggucagugcugcu......................................................................  930035  0  seq
....................cucaagugUggucagugcugcu......................................................................  70      1  seq
....................cucGagugaggucagugcugcu......................................................................  281     1  seq
....................cucaagugagguAagugcugcu......................................................................  216     1  seq
....................cucaagugagAucagugcugcu......................................................................  128     1  seq
....................cucaagugagguGagugcugcu......................................................................  103     1  seq
....................cucaagugaggucagugcugNu......................................................................  2       1  seq
....................cucaaguAaggucagugcugcu......................................................................  902     1  seq
....................cucaagugaggGcagugcugcu......................................................................  39      1  seq
....................cucaagugaggucagugUugcu......................................................................  66      1  seq
....................cucaagugaAgucagugcugcu......................................................................  516     1  seq
....................cAcaagugaggucagugcugcu......................................................................  1089    1  seq
....................cucaagugaggucagGgcugcu......................................................................  70      1  seq
....................cucaagugaggucagugcugcG......................................................................  816     1  seq
....................cuUaagugaggucagugcugcu......................................................................  537     1  seq
....................cucaagugaggucagugcuUcu......................................................................  66      1  seq
....................cucaagugaggucagugAugcu......................................................................  177     1  seq
....................cucaUgugaggucagugcugcu......................................................................  53      1  seq
....................cucaagugaggucagugcuAcu......................................................................  109     1  seq
....................cucaagugaggucUgugcugcu......................................................................  98      1  seq
....................cucaagugaggucagugcAgcu......................................................................  226     1  seq
....................cucaaguCaggucagugcugcu......................................................................  84      1  seq
....................cucaGgugaggucagugcugcu......................................................................  128     1  seq
....................cucaagugaUgucagugcugcu......................................................................  59      1  seq
....................Aucaagugaggucagugcugcu......................................................................  291     1  seq
```

```
                    Mature                Star
uuugucuagcgucucgcugucucaagugaggucagugcugcuguguuuucuaacaggggcacugucuucaauugaaaccguuggaccggaacucaccaucaauuucacuuuc
....................cucaagugGggucagugcugcu......................................................................    115   1   seq
....................cucaagCgaggucagugcugcu......................................................................    219   1   seq
....................cucaagugaggAcagugcugcu......................................................................    458   1   seq
....................cucaagugaggucaguAcugcu......................................................................    88    1   seq
....................cucaagugagCucagugcugcu......................................................................    57    1   seq
....................cucaagugaggucagugcugcA......................................................................    742   1   seq
....................cucaagugCggucagugcugcu......................................................................    12    1   seq
....................cucaagugaggucaguCcugcu......................................................................    50    1   seq
....................cucaagugaggCcagugcugcu......................................................................    97    1   seq
....................cucaagugaggucagugGugcu......................................................................    79    1   seq
....................cucaaAugaggucagugcugcu......................................................................    67    1   seq
....................cuGaagugaggucagugcugcu......................................................................    46    1   seq
....................cGcaagugaggucagugcugcu......................................................................    73    1   seq
....................cucaagugaNgucagugcugcu......................................................................    2     1   seq
....................cucCagugaggucagugcugcu......................................................................    21    1   seq
....................cucaagugaggucagugcugGu......................................................................    88    1   seq
....................cucUagugaggucagugcugcu......................................................................    45    1   seq
....................Gucaagugaggucagugcugcu......................................................................    39    1   seq
....................cucaagugaggucagAgcugcu......................................................................    1006  1   seq
....................cucaagugaggucagugcGgcu......................................................................    86    1   seq
....................cucaagugaggucagCgcugcu......................................................................    121   1   seq
....................cCcaagugaggucagugcugcu......................................................................    128   1   seq
....................cuNaagugaggucagugcugcu......................................................................    3     1   seq
....................cucaagugaggucagugcugUu......................................................................    64    1   seq
....................cucaagugaggucaUugcugcu......................................................................    44    1   seq
....................cucaagugaggucagugcuCcu......................................................................    63    1   seq
....................cucaaguUaggucagugcugcu......................................................................    96    1   seq
....................cucaagugagUucagugcugcu......................................................................    69    1   seq
....................cucaagugaggucagugcugcN......................................................................    1     1   seq
....................cucaagugaggucaguUcugcu......................................................................    75    1   seq
....................cucaagugaggucagugcugAu......................................................................    232   1   seq
....................cucaagugaggucaAugcugcu......................................................................    71    1   seq
....................cucaagugaggucaCugcugcu......................................................................    31    1   seq
....................cucaagugaggucagugcugcC......................................................................    206   1   seq
....................cucaagugaCgucagugcugcu......................................................................    53    1   seq
....................cucaCgugaggucagugcugcu......................................................................    8     1   seq
....................cucaagugagguUagugcugcu......................................................................    301   1   seq
....................cucaagGgaggucagugcugcu......................................................................    177   1   seq
....................Nucaagugaggucagugcugcu......................................................................    280   1   seq
....................cucaagugaggucCgugcugcu......................................................................    19    1   seq
....................cucaagAgaggucagugcugcu......................................................................    1211  1   seq
....................cucaagugaggucagugcCgcu......................................................................    138   1   seq
....................Uucaagugaggucagugcugcu......................................................................    600   1   seq
....................cuAaagugaggucagugcugcu......................................................................    421   1   seq
....................cucaaCugaggucagugcugcu......................................................................    66    1   seq
....................cucaagugaggucGgugcugcu......................................................................    151   1   seq
....................cucaaUugaggucagugcugcu......................................................................    65    1   seq
....................cNcaagugaggucagugcugcu......................................................................    39    1   seq
....................Aucaagugaggucagugcugcug.....................................................................    1     1   seq
....................cucaagugaggucagugcugcug.....................................................................    3022  0   seq
....................cucaagugaggucagugcuAcug.....................................................................    1     1   seq
....................cucaagugaggucagugcugcuA.....................................................................    1191  1   seq
....................cucaagugaggucagugcugcuU.....................................................................    8486  1   seq
....................cucaagugagguAagugcugcug.....................................................................    3     1   seq
....................cucaagugaggucagugcugcGg.....................................................................    2     1   seq
....................cucaagugaggucagugcugcAg.....................................................................    5     1   seq
....................cucaagugaggucagugcugGug.....................................................................    1     1   seq
....................cucaagugaggAcagugcugcug.....................................................................    1     1   seq
....................cucaagugaggucagugcAgcug.....................................................................    1     1   seq
....................Nucaagugaggucagugcugcug.....................................................................    2     1   seq
....................cucaagugaggucagugcuUcug.....................................................................    1     1   seq
....................cucaagugaggucagugcugcuC.....................................................................    66    1   seq
....................cuAaagugaggucagugcugcug.....................................................................    3     1   seq
....................cucaaAugaggucagugcugcug.....................................................................    1     1   seq
....................cucaagugagguGagugcugcug.....................................................................    1     1   seq
....................cucaaguAaggucagugcugcug.....................................................................    2     1   seq
....................cuUaagugaggucagugcugcug.....................................................................    2     1   seq
....................cucaagugagguUagugcugcug.....................................................................    1     1   seq
....................cucaagugagAucagugcugcug.....................................................................    2     1   seq
....................cucaagugaggucagugcugAug.....................................................................    1     1   seq
```

```
                    Mature                            Star
uuugucuagcgucucgcugucucaagugaggucagugcugcuguguuuucuaacaggggcacugucuucaauugaaaccguuggaccggaacucaccaucaauuucacuuuc
....................cucaagugaggucagAgcugcug.....................................................................  4     1  seq
....................cucaagugaggucagugcugcCg.....................................................................  1     1  seq
....................cucaagugaUgucagugcugcug.....................................................................  1     1  seq
....................cucaagugaggucagugcugcuN.....................................................................  1     1  seq
....................cucaagugGggucagugcugcug.....................................................................  1     1  seq
....................cucaagugaAgucagugcugcug.....................................................................  1     1  seq
....................cucGagugaggucagugcugcug.....................................................................  1     1  seq
....................cucUagugaggucagugcugcug.....................................................................  2     1  seq
....................Uucaagugaggucagugcugcug.....................................................................  1     1  seq
....................cucaaguUaggucagugcugcug.....................................................................  1     1  seq
....................cAcaagugaggucagugcugcug.....................................................................  6     1  seq
....................cucaagAgaggucagugcugcug.....................................................................  4     1  seq
....................cucaagugaggucagugcugcuCu....................................................................  4     1  seq
....................cucaagugaggGcagugcugcugu....................................................................  1     1  seq
....................cucaagugaggucagugcugcuUu....................................................................  1001  1  seq
....................cucaagugaggucagugcugcugA....................................................................  11    1  seq
....................cucaagugaggucagugcugcugu....................................................................  220   0  seq
....................cucaagugaggucagugcugcuAu....................................................................  229   1  seq
....................cAcaagugaggucagugcugcugu....................................................................  1     1  seq
....................cucaagugaggucagugcugcugG....................................................................  1     1  seq
....................Uucaagugaggucagugcugcugu....................................................................  2     1  seq
....................cucaagugaggucagugcugcuUug...................................................................  2     1  seq
....................cucaagugaggucagugcugcuguU...................................................................  17    1  seq
....................cucaagugaggucagugcugcuguUu..................................................................  4     1  seq
....................cucaagugaggucagugcugcuguAuu.................................................................  1     1  seq
....................cucaagugaggucagugcugcuguUuu.................................................................  3     1  seq
.....................ucaagugaggucagugcu.........................................................................  1     0  seq
.....................ucaGgugaggucagugcu.........................................................................  1     1  seq
.....................ucaagugaggucagugcugc.......................................................................  110   0  seq
.....................ucaagAgaggucagugcugcu......................................................................  2     1  seq
.....................ucaagugaggucagugcuAcu......................................................................  1     1  seq
.....................Ccaagugaggucagugcugcu......................................................................  2     1  seq
.....................ucaagugaUgucagugcugcu......................................................................  1     1  seq
.....................ucGagugaggucagugcugcu......................................................................  1     1  seq
.....................ucaGgugaggucagugcugcu......................................................................  1     1  seq
.....................ucaaguAaggucagugcugcu......................................................................  1     1  seq
.....................ucaagugaggucagugcugcG......................................................................  2     1  seq
.....................Gcaagugaggucagugcugcu......................................................................  2     1  seq
.....................ucaagugagguAagugcugcu......................................................................  3     1  seq
.....................ucaagugaggucagugcAgcu......................................................................  1     1  seq
.....................ucaagugaAgucagugcugcu......................................................................  3     1  seq
.....................ucUagugaggucagugcugcu......................................................................  1     1  seq
.....................ucaagugagCucagugcugcu......................................................................  1     1  seq
.....................ucaagugCggucagugcugcu......................................................................  2     1  seq
.....................ucaagugaggucUgugcugcu......................................................................  1     1  seq
.....................Ncaagugaggucagugcugcu......................................................................  1     1  seq
.....................uAaagugaggucagugcugcu......................................................................  9     1  seq
.....................ucaagugaggucaguAcugcu......................................................................  1     1  seq
.....................uUaagugaggucagugcugcu......................................................................  2     1  seq
.....................Acaagugaggucagugcugcu......................................................................  9     1  seq
.....................ucaagugaggucagugcugAu......................................................................  2     1  seq
.....................ucaagugaggucagugAugcu......................................................................  3     1  seq
.....................ucaagugaggucGgugcugcu......................................................................  1     1  seq
.....................ucaagugaggucaUugcugcu......................................................................  1     1  seq
.....................ucaagugaggucagugcugcu......................................................................  2838  0  seq
.....................ucaagugaggucagugcugcuU.....................................................................  12    1  seq
.....................ucaagugaggucagugcugcug.....................................................................  17    0  seq
.....................ucaagugaggucagugcugcuA.....................................................................  7     1  seq
.....................ucaagugaggucagugcugcuUu....................................................................  5     1  seq
.....................ucaagugaggucagugcugcugu....................................................................  3     0  seq
......................caagugaggucagugcugcu......................................................................  39    0  seq
......................caagugaggucagugcugcugu....................................................................  8     0  seq
.......................aagugaggucagugcugc.......................................................................  1     0  seq
.......................aagugaggucagugcugcu......................................................................  19    0  seq
........................aguAaggucagugcugcu......................................................................  1     1  seq
........................Ggugaggucagugcugcu......................................................................  1     1  seq
........................agugaggucagugcugcu......................................................................  63    0  seq
.......................................................ggggcacugucuucaauugaaa...................................  25    0  seq
.......................................................ggggcacugucuucaauugaaac..................................  299   0  seq
.......................................................Agggcacugucuucaauugaaac..................................  1     1  seq
```

```
                    Mature                          Star
uuugucuagcgucucgcugucucaagugaggucagugcugcuguguuuucuaacaggggcacugucuucaauugaaaccguuggaccggaacucaccaucaauuucacuuuc
.......................................................ggggcacuguAuucaauugaaac..................................  1    1    seq
.......................................................ggggcacuCucuucaauugaaac..................................  1    1    seq
.......................................................gggAcacugucuucaauugaaac..................................  1    1    seq
.......................................................ggggcacugucuucaauugaaacU.................................  29   1    seq
........................................................gggcacugucuucaauugaa....................................  2    0    seq
........................................................gggcacugucAucaauugaaa...................................  1    1    seq
........................................................gggcacugucuucaauugaaa...................................  3    0    seq
........................................................gggcacugucuucaauuAaaac..................................  3    1    seq
........................................................gggcacugucuuAaauugaaac..................................  1    1    seq
........................................................gggcacugAcuucaauugaaac..................................  2    1    seq
........................................................gggcacugucuucaauugaaaA..................................  1    1    seq
........................................................gggcacugucuucaauugaGac..................................  1    1    seq
........................................................gggcacugucuuUaauugaaac..................................  1    1    seq
........................................................gggcacugucuucaauugaaac..................................  833  0    seq
........................................................gAgcacugucuucaauugaaac..................................  2    1    seq
........................................................Uggcacugucuucaauugaaac..................................  1    1    seq
........................................................gggcacugucAucaauugaaac..................................  1    1    seq
........................................................Aggcacugucuucaauugaaac..................................  2    1    seq
........................................................gggcaUugucuucaauugaaac..................................  1    1    seq
........................................................gggcacugucuucaauugaaacA.................................  2    1    seq
........................................................gggcacugucuucaauugaaacU.................................  25   1    seq
.........................................................ggcacugucuucaauugaaa...................................  1    0    seq
..............................................................................cguuggaccggaacucac................  9    0    seq
..............................................................................cguuggaccggaacucaA................  2    1    seq
..............................................................................cguuggaccggaacucacc...............  12   0    seq
..............................................................................cguuggaccggaacucacA...............  1    1    seq
..............................................................................cguuggaccggaacucacca..............  7    0    seq
..............................................................................cguuggaccggaacucacAa..............  3    1    seq
..............................................................................Uguuggaccggaacucaccau.............  1    1    seq
..............................................................................cguuAgaccggaacucaccau.............  1    1    seq
..............................................................................cguuggaccggaacucaccau.............  4    0    seq
..............................................................................cguuggaccggaacucaccaA.............  4    1    seq
..............................................................................cguuggaccggaacucaccauca...........  2    0    seq
..............................................................................cguuggaccggaacucaccaucaau.........  1    0    seq
..............................................................................cguuggaccggaacucaccaucaauu........  3    0    seq
..............................................................................cguuggaccggaacucaccaucaauuA.......  1    1    seq
..............................................................................cguuggaccggaacucaccaucaauuuA......  6    1    seq
..............................................................................cguuggaccggaacucaccaucaauuuca.....  1    0    seq
..............................................................................cguuggaccggaacucaccaucaauuuAa.....  7    1    seq
..............................................................................cguuggaccggaacucaccaucaauuucac....  1    0    seq
..............................................................................cguuggaccggaacucaccaucaauuucaA....  3    1    seq
..............................................................................cguuggaccggaacucaccaucaauuucacu...  2    0    seq
```
